# Colored Light Exposure Ensues Chronotype-Based Responses: Evidence From QEEG Analysis

**DOI:** 10.33549/physiolres.935548

**Published:** 2025-06-01

**Authors:** Vakode VANI, Pooja OJHA, Mahesh Arjundan GADHVI, Abhinav DIXIT

**Affiliations:** 1 Department of Physiology, All India Institute of Medical Sciences, Jodhpur, Rajasthan, India

**Keywords:** Circadian rhythm, Color of light, Light and chronotype, Wavelength of light, EEG in chronotypes

## Abstract

Light spectra, an imperative zeitgeber, may differ in its chronobiological effects among chronotype ensuing differences in circadian pacesetting. With the increasing usage of colored lights in the environment, the effects of light wavelength on the electrical activity of the brain among chronotypes need to be investigated. Healthy participants (N=24) were recruited to morning, intermediate, and evening chronotype groups using the composite scale for morningness scores. They were exposed to randomized brief sessions of red, green, blue, and white light preceded by 15 min of darkness. EEG was recorded in all sessions. The power spectrum was estimated for alpha, beta, theta, and delta waves from different regions of the scalp and compared among the groups. The morning and evening chronotype had statistically significantly higher mean delta power than intermediate chronotype in colored light. Evening chronotype showed a statistically significantly higher mean beta power than the intermediate chronotype (p=0.013) in green light. Intermediate chronotype had statistically significantly higher mean alpha power than morning (p=0.029) and evening chronotype (p=0.009) in red light. The results show a significant effect of the spectral property of light on brain waves in chronotypes. The green light is more effective in alerting evening chronotypes. The finding of the present study may be applicable in research pertinent to brain imaging in chronotypes especially with red, green, and blue light exposure and chromotherapy-based interventions in affective and psychiatric conditions.

## Introduction

The circadian functions entrain to the solar day-night rhythm through daily environment signals. The entrainment with the day-night cycle could be either earlier or late ensuing a natural interindividual difference in chronotypes that defines an individual’s physiological, behavioral, and cognitive tunings over a 24-h solar day [1]. An essential factor that contributes to the observed differences among chronotypes is a differing “zeitgeber” signal providing clues to the extrinsic solar rhythm. The light-dark cycle comprises an essential zeitgeber for living beings, including humans and it has been reported that chronotype correlates with the duration of daily exposure to bright light [1–3]. In addition, the nature and time of light exposure have a significant effect on the entrainment. Natural light has been found to advance the circadian rhythm compared to artificial light [4]. Exposure to morning light result in advanced circadian phase while evening light exposure delays the circadian phase [5].

Contemplatively, Light spectra may vary in its chronobiological actions on the suprachiasmatic nucleus (SCN), the circadian pacesetter [6,7]. The SCN of the hypothalamus modulates the phase by the spectral attributes of the ambient light levels and communicates the same to the peripheral organs and cells through the autonomic and humoral mediators [8]. The chronobiological actions of light on the SCN may be attributed to the composition of light spectra in the environment at dawn and dusk, possibly an increment in the shorter wavelength than the mid-long wavelength. Of note, the spectral sensitivity of circadian phase shifting is reportedly close to the peak spectral sensitivity for melanopsin containing intrinsically photosensitive retinal ganglion cells though cones have also been found to contribute to the non-visual effects of light [9,10]. The circadian system likely compares the outputs from different photoreceptors to estimate the spectral composition of light and thereby timing of dawn and dusk [11]. The effects of daytime exposure to short-wavelength light has been found to be associated with increased evening fatigue, decreased sleep-onset latency, and higher slow-wave sleep accumulation [12–14].

Electroencephalogram has provided a non-invasive tool for assessment of brain functions. The spontaneous electrical activity of the brain spans over several frequencies that have been designated as delta, theta, alpha, beta. The frequencies of EEG waves ranges from 0.5 to 4 Hz for delta waves, 4 to 8 Hz for theta wave, 7.5 to 13 Hz for alpha waves, and from 12 to 35 Hz for beta waves [15]. With the help of quantitative analysis of EEG rhythm, the power changes in various brain regions can be estimated and compared. EEG rhythms, especially alpha and beta, have been found to show region and task-dependent circadian rhythmicity with a peak in the afternoon [16]. Reflectively, EEG activity phase is likely to vary with chronotype as they differ in behavior and alertness across the day [17].

The effects of light exposure on EEG during evening hours and before bedtime have been investigated. Exposure to short-wavelength light 3 h before bedtime shows a reduction in slow wave activity and causes shallow sleep [18]. However, the differences in EEG responses to narrow-spectrum light as a function of chronotype has remained limited. The findings will be informative for planning and designing customized ambient lighting environments for different chronotypes as chronotype affects physical and mental functions. It will offer compelling ground for complimentary color-based therapy in affective disorders that are linked to circadian rhythm [19]. Therefore, this work is planned to explore the brain wave responses to light exposure of varying wavelengths during daytime among chronotypes.

## Material and Methods

This prospective interventional study was conducted in the EEG laboratory during daytime. The study commenced after approval from the Institutes Ethical committee was sought (AIIMS/IEC/2022/4149). Healthy individuals in the age range 18–45 years were considered for the study. Participants completed the Composite Scale of Morningness Questionnaire (CSM). The CSM questions were asked to assess participant’s chronotype, preferred daytime hours, sleeping patterns, and sleep-wake cycle. Individuals with a CSM score higher than 49 were classified as morning types. Intermediates had a score between 36 and 49, and evening types had a score below 36. Mini-Mental Status Examination (MMSE) scores were obtained for each participant. MMSE included eleven questions that assessed comprehension, communication, reasoning, and memory. Participants with MMSE scores between 26 to 30 were enrolled in the study. Individuals with color blindness, photophobia, history of neurological disease, intake of the drug causing sedation, sleep disturbance, night shift workers, and history of recent trans-meridian travel were excluded from the study. Written informed consent was obtained from all participants.

The procedure was explained to the participants and they were instructed to avoid consumption of tea, coffee, and smoking on the day of recording. All recordings were conducted between 10:00–12:00 h.

The experiment comprised four sessions in which the participants were exposed to four light conditions: white, green, red, and blue lights, separately using GANIT RGB LED FLOOD LIGHT. The four light sessions, constituting white light, red light (wavelength 612 nm), green light (wavelength 548 nm), and blue light (wavelength 464 nm), were counterbalanced and conducted in randomized order. The light was placed overhead the participant. Each light session was preceded by 15 min of the dark state. EEG was recorded for 5 mins using RMS Superspec 32 equipment, and 19 channels were used.

### Recording of the Electroencephalogram

The participants were seated comfortably. The dimensions of the head in antero-posterior and transverse directions were measured and electrode positions were marked according to the international 10–20 system. The scalp was prepared using skin prep gel before applying the electrodes with the help of EEG conductive paste. The impedance was checked and kept below 10 kΩ. The participants were instructed to relax, look straight at a fixed point on the wall, and limit their body movements during EEG recording. In each light session, EEG was recorded for 5 min. EEG data was analyzed using a fast Fourier transform. A band pass filter with a frequency range of 1–70 Hz was employed to exclude signals that fall beyond the needed frequency range. The EEG recording devoid of eye blinks, body movements, eyeball movements, and other artifacts were selected for analysis. The data were subsequently organized into epochs of two seconds each. The EEG data of each epoch was exported to an Excel sheet. In each light session, from the 5-minute EEG recording, ten epochs with a total duration of 20 s were randomly selected and used for analysis. The average power spectra of alpha, beta, theta, and delta waves were obtained using all the epochs.

**Fig. 1:**
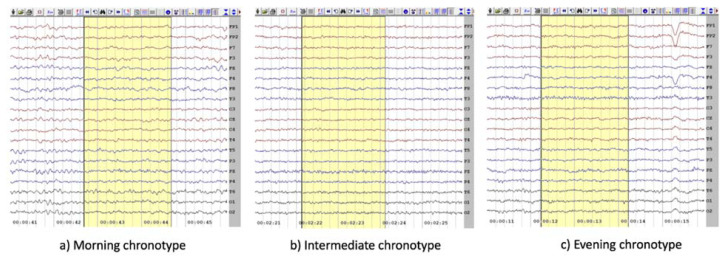
This Figure shows the sample of EEG recording from (**a**) Morning, (**b**) Intermediate, and (**c**) Evening chronotype groups.

The power recorded from Fp1, Fp2, F7, F8, F3, F4, Fz, C3, C4, and Cz electrodes were averaged and mentioned as power in the frontocentral region. The power averaged from the electrodes P3, P4, and Pz was noted as power in the parietal region. The power recorded from the T3, T4, T5, and T6 electrodes were averaged and noted as power in the temporal region. The power averaged from the electrodes O1 and O2 were mentioned as power in the occipital region. Sample of EEG recording from each chronotype group is presented in Figure 1.

### Statistical tests

Data was analyzed using SPSS software. The continuous and categorical data have been presented as mean and numbers respectively. The EEG powers in three groups have been compared using Analysis of variance with a *post hoc* test to look for inter-group comparison. A p value less than 0.05 was considered statistically significant.

## Results

Twenty-four males participated in the study. The participants were recruited in morning (n=8), intermediate (n=8), and evening chronotype (n=8) groups according to their CSM scores having a mean (standard deviation) age of 28.25 (2.71), 28 (5.73), and 27.38 (3.85) years respectively. The mean (standard deviation) MMSE score of morning, intermediate and evening chronotypes was 28.75 (1.03), 29.38 (1.4), and 28.88 (0.99) respectively. There was no statistically significant difference in age (p=0.916) and MMSE score (p=0.532) among the groups.

The mean relative powers of alpha, beta, theta, and delta waves in frontocentral, parietal, temporal, and occipital regions during four randomized sessions with red, green, blue, and white light were obtained. A one-way ANOVA determined a statistically significant difference in mean relative powers of alpha waves (F_(2,21)_=4.637, p=0.021) and delta waves (F_(2,21)_=4.165, p=0.030) in the parietal region with exposure to red light between the chronotypes. In addition, a Tukey *post hoc* test showed that with red light exposure intermediate chronotype had statistically significantly higher mean alpha power than morning (p=0.029) and evening chronotype (p=0.009). The intermediate chronotype also had statistically significantly lower mean delta power in parietal regions than the morning (p=0.032) and evening chronotype (p=0.015).

With the green light, a statistically significant difference in mean relative powers of beta waves in the temporal (F_(2,21)_=3.798, p=0.039) and delta wave in the parietal (F_(2,21)_=3.909, p=0.036) region was found. Tukey *post hoc* test showed that the evening chronotype had statistically significantly higher mean beta power in the temporal region than the intermediate chronotype (p=0.013) whereas the intermediate chronotype had statistically significantly lower mean delta power in the parietal region than morning (p=0.015) and evening chronotype (p=0.049).

With blue light exposure, a statistically significant difference in mean relative powers of delta waves in the parietal (F_(2,21)_=4.545, p=0.023) region was found. Tukey *post hoc* test showed that the intermediate chronotype had statistically significantly lower mean delta power in parietal regions than the morning (p=0.037) and evening (p=0.009) chronotype.

**Fig. 2:**
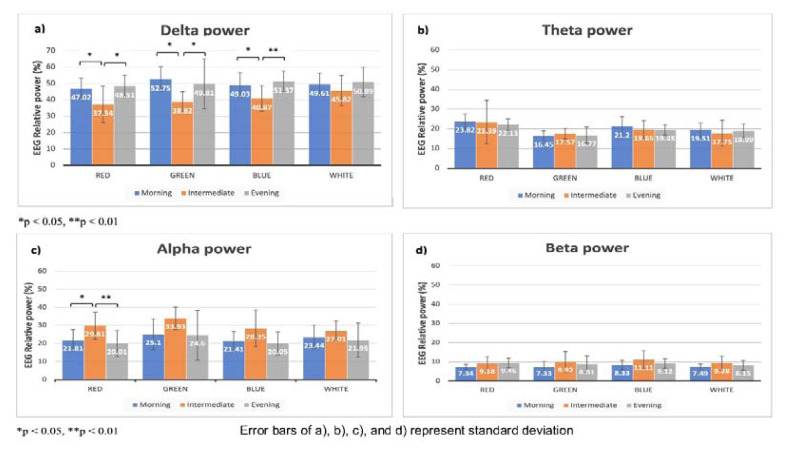
Comparison of mean relative powers of EEG waves in four lights in parietal region.

**Fig. 3:**
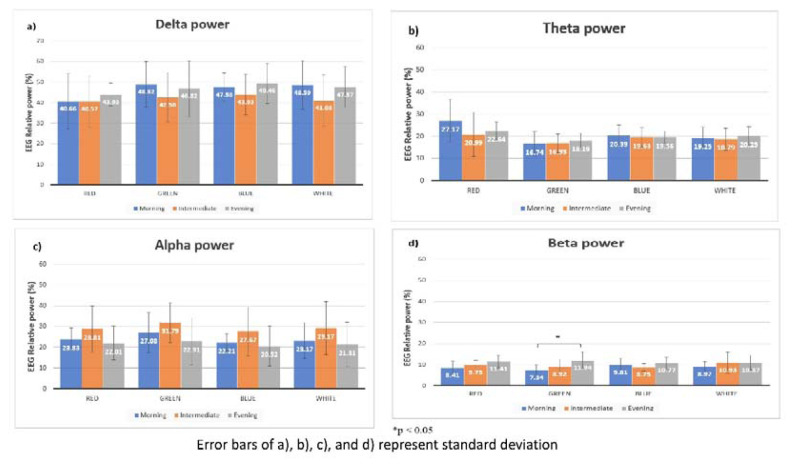
Comparison of mean relative powers of EEG waves in four lights in Temporal region.

In contrast, there was no statistically significant difference in EEG power with white light exposure among the three chronotype groups (p>0.05). Figures 2 and 3 show the differences in mean relative powers of four waves in four lights in parietal and temporal regions respectively.

There was no statistically significant difference in EEG power in four lights among the three chronotype groups in frontocentral and occipital regions. The data has been presented in Table 1.

## Discussion

The present study was conducted to investigate whether exposure to monochromatic light produces distinct responses in EEG rhythm among chronotypes. The main outcome of this work suggests that chronotypes are differentially sensitive to light frequencies. We found that evening chronotypes exhibit higher cortical activation depicted with an increase in beta power with daytime exposure to green light whereas with red and blue light they show enhanced delta power.

Previous studies exploring the effects of daytime exposure to monochromatic light have used blue or red color light and compared the changes in EEG waves with polychromatic white light or darkness [20–22]. The study of Sahin and Figueriro suggested an alerting effect of red light with a reduction in alpha and theta power when compared to darkness. They did not find a significant alerting effect of blue light on EEG waves [20]. In contrast, Iskra-Golec demonstrated that the alerting effects of monochromatic blue light exposure were seen with short 30-minute exposure only. Longer exposure of 4 h had a reverse effect with an increase in EEG theta and alpha power [21]. They did not explore the effects of chronotypes on the EEG response in this work. In a recent study investigating the effects of blue light on chronotypes, the morning chronotypes were found to have higher theta and alpha power in the afternoon hours compared to polychromatic white light in the morning or evening hours. No such effect was observed in the evening chronotypes [22]. The authors suggested a decline in alertness among the morning chronotypes.

Based on the results presented here, there seems to be a definite effect of chronotype on EEG waves with exposure to red, green, and blue lights suggesting differences in susceptibility to light color among chronotypes. The differences in response of morning, intermediate, and evening chronotypes may be cogitated through the growing body of evidence supporting structural and functional differences in the brain comprising differential intrinsic phase setting, light sensitivity, and cyclical changes in alertness among the chronotypes across the day [23–25]. The morning chronotype is related to lower regional gray matter density in the left posterior parietal cortex, and precuneus, and higher gray matter density in both the orbitofrontal cortex and hypothalamic regions around the suprachiasmatic nucleus [26]. Magnetic resonance imaging-based research revealed an association of evening chronotype with localized right hippocampus atrophy in the mid-anterior region that did not correlate with age, gender, quality of sleep, and mood [27]. Additionally, in a recent study, a greater volume of the left anterior occipital sulcus was reported in evening chronotypes [28]. The differences in the visual cortex may probably reflect the differences in communicating light signals to the circadian system. Interestingly, differences in the corpus callosum, cingulate gyrus, and frontal and temporal lobe in the evening chronotype compared to intermediate and morning types have also been reported [29]. Reports also suggest differences in functional connectivity of the default mode network that plays a vital role in emotion processing, attentional control, working memory, and consciousness awakening between evening and morning chronotype individuals [30]. An important difference in the morning and evening chronotype is the intrinsic circadian period that may result from a single mutation in the clock gene [31]. The period of the circadian oscillator is not exactly 24 h and hence necessitates regular zeitgeber [32]. A shorter intrinsic circadian period is related to morning types having a faster biological clock with phase advancement. On the contrary, a long intrinsic period with phase delay leads to evening chronotypes [33]. Contemplatively, zeitgeber plays a crucial role in coordinating the circadian oscillator and variability in the circadian phase [34].

Light, an important zeitgeber, regulates several physiological functions through the intrinsically photosensitive retinal ganglion cells, besides vision [35]. The phase response curve, essentially reflects the relation between the time of light exposure and phase shift of the circadian rhythm [36]. Daily differences in exposure to light of varying spectral composition, among chronotypes, probably result in a differential sensitivity to various monochromatic bands of light.

Present study shows a higher delta power among morning and evening chronotypes in red, green, and blue lights compared to intermediate chronotype. Oscillations in delta band have been implicated in higher emotional engagement, motivational processes pertinent with reward and fear, and many cognitive processes [37–39]. Delta wave has been found to enhance proficiency in task accomplishment. A relatively paradoxical relation between increased delta wave and mental task has been attributed to a possible selective neural suppression of unrelated signal by delta oscillation during the execution of mental activity. The higher delta power among morning and evening chronotype in colored lights possibly reveals a distinct effect of light spectrum on non-image forming functions of light in chronotype.

**Fig. 4:**
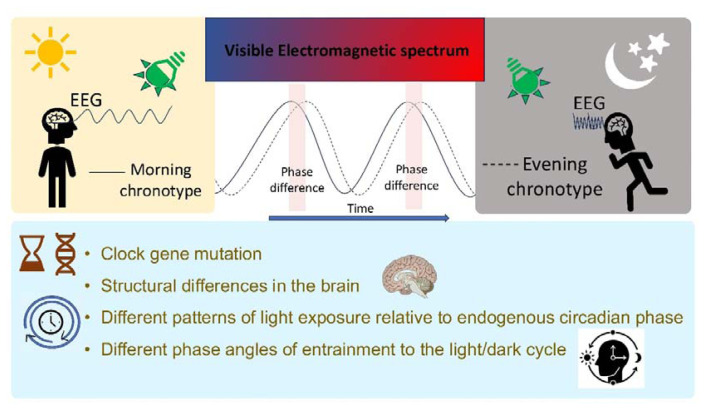
Possible mechanisms accounting for differences brain activity in colored light among chronotypes.

Conceivable mechanisms accounting for differences in morning and evening chronotypes have been presented in Figure 4.

Besides, white light exposure has shown no statistical difference among the chronotypes whereas the red, green, and blue lights have effectively demonstrated differences in brain responses to light among the chronotypes. The results of the study have yielded essential insight into light and chronotype responses. However, we have not enrolled female participants limiting the extrapolation of its results on them.

Conclusively, the color of light as well as circadian clock rhythm have an interaction. This interaction results in differential cortical electrical activity as recorded with the EEG. Intermediate chronotypes had higher cortical activation with red and blue light compared to morning and evening chronotypes. The evening chronotype had higher beta power with green light. Chronotypes should be determined in research related to light exposure, especially with exposure to red, green, and blue lights. The finding of the present study may be applicable in research pertinent to occupations related with working in colored light environment.

## Figures and Tables

**Table 1:** EEG relative power in frontocentral and occipital regions in four lights among the chronotype.

Light Color	EEG Wave	Chronotype	Frontocentral Region			Occipital Region		

			Mean Relative Power	SD	p value	Mean Relative Power	SD	p value
WHITE	Alpha	Morning	19.08	4.75	p=0.15	25.99	9.52	p=0.39
		Intermediate	24.07	6.61		27.41	4.98	
		Evening	18.52	6.58		22.08	8.42	

	Beta	Morning	7.50	2.59	p=0.59	8.36	2.34	p=0.26
		Intermediate	8.33	3.22		8.46	2.11	
		Evening	9.01	2.95		10.25	2.96	

	Theta	Morning	20.37	2.83	p=0.37	20.24	4.21	p=0.75
		Intermediate	17.99	4.88		21.92	5.10	
		Evening	20.32	3.31		21.33	4.25	

	Delta	Morning	53.62	5.76	p=0.32	45.49	10.51	p=0.62
		Intermediate	48.87	6.92		42.19	5.72	
		Evening	52.36	6.37		46.34	9.92	

RED	Alpha	Morning	19.59	3.01	p=0.09	22.47	5.98	p=0.16
		Intermediate	23.92	6.37		27.41	8.96	
		Evening	18.06	5.79		19.76	7.82	

	Beta	Morning	7.59	1.73	p=0.45	8.29	1.12	p=0.22
		Intermediate	9.65	5.03		8.05	4.38	
		Evening	9.36	2.84		10.70	3.39	

	Theta	Morning	26.63	4.65	p=0.51	25.87	4.82	p=0.80
		Intermediate	24.40	8.50		24.38	10.15	
		Evening	23.06	4.25		23.40	6.37	

	Delta	Morning	46.17	6.88	p=0.14	43.37	8.32	p=0.42
		Intermediate	42.02	9.00		40.15	10.30	
		Evening	49.50	5.00		46.14	8.08	

GREEN	Alpha	Morning	21.72	6.30	p=0.15	26.55	9.46	p=0.36
		Intermediate	26.98	5.55		32.36	8.79	
		Evening	20.45	8.07		24.82	13.53	

	Beta	Morning	6.51	1.50	p=0.21	7.45	1.87	p=0.42
		Intermediate	8.41	4.12		10.04	6.27	
		Evening	9.49	3.67		10.11	4.23	

	Theta	Morning	19.23	3.95	p=0.82	16.48	6.19	p=0.75
		Intermediate	18.34	4.59		18.68	5.98	
		Evening	18.03	3.51		18.03	5.69	

	Delta	Morning	52.53	8.20	p=0.25	49.50	12.02	p=0.18
		Intermediate	46.11	7.57		38.77	6.89	
		Evening	52.30	9.68		47.03	15.00	

BLUE	Alpha	Morning	19.44	4.17	p=0.16	21.90	4.85	p=0.25
		Intermediate	21.76	6.78		27.97	10.58	
		Evening	16.40	4.68		21.51	8.78	

	Beta	Morning	7.65	2.59	p=0.54	10.16	2.59	p=0.57
		Intermediate	9.49	4.54		9.71	2.91	
		Evening	9.18	3.04		11.42	4.19	

	Theta	Morning	20.07	3.08	p=0.87	19.13	5.56	p=0.72
		Intermediate	19.51	3.86		21.36	6.93	
		Evening	19.30	2.15		20.88	4.93	

	Delta	Morning	52.89	4.22	p=0.16	48.78	5.86	p=0.06
		Intermediate	49.19	7.64		40.33	5.52	
		Evening	55.10	5.66		46.17	8.40	
